# Physiology of Pacing Symposium 2024: Letter from the Program Directors

**DOI:** 10.19102/icrm.2024.15125

**Published:** 2024-12-15

**Authors:** Pugazhendhi Vijayaraman, Gopi Dandamudi, Kenneth A. Ellenbogen, Gaurav Upadhyay, Roderick Tung

**Affiliations:** Geisinger Heart Institute, Wilkes Barre, PA, USA; Prisma Health, Greenville, SC, USA; Virginia Commonwealth University Health System, Richmond, VA, USA; University of Chicago Medicine, Chicago, IL, USA; Arizona College of Medicine, Banner Health, Phoenix, AZ, USA

**Keywords:** Cardiac pacing, conference, left bundle branch, His bundle


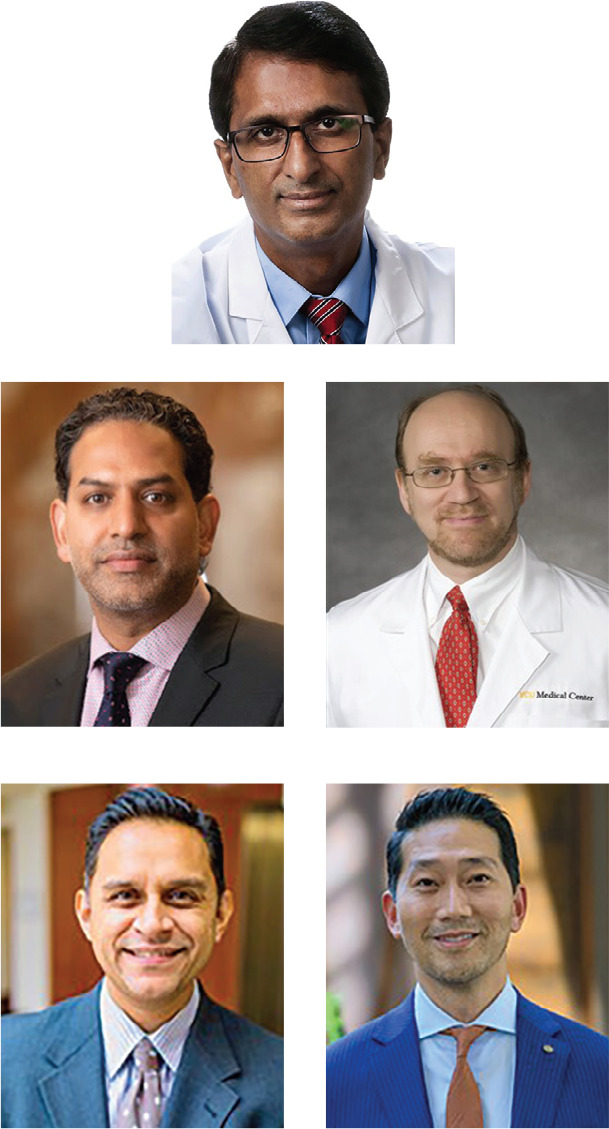


Dear Readers,

As the Program Directors for the Eighth Annual Physiology of Pacing Symposium, we are pleased to present the “Top 3” original research abstracts selected from the many interesting scientific research abstracts in the field of pacing submitted to our annual symposium. After a thorough review of the numerous exceptional scientific abstracts submitted by physicians from across the globe, the program committee chose these finalists to present their work and participate in panel discussions, with the overall winner announced at the sessions’ conclusion following the panel deliberation. (For those who were unable to attend or who wish to view the sessions again, the Eighth Annual Physiology of Pacing Symposium ON-DEMAND at www.innovationsincrm.com/pps2024 provides instant and unlimited access to the full library of educational programming from the Symposium.)

Dr. Kielbasa et al., from Jagiellonian University Medical College in Krakow, Poland, presented the results of their research on the value of using global R-wave peak time (RWPT) to assess left bundle branch (LBB) capture. They determined that combining RWPTs in leads I and V6 provided greater sensitivity and specificity to accurately identify LBB capture during LBB area pacing. This new criterion is minimally influenced by the QRS axis or rS morphology in lead V6.

Dr. Ponnusamy et al., from Velammal Medical College Hospital and Research Institute in Madurai, India, presented their new insights from the prospective LOCAlizaTion and Clinical CorrElation of Left Bundle Branch Pacing lead computed tomographic angiography (LOCATE-LBBP) study. They analyzed paired cardiac computed tomography angiography both after LBB pacing (LBBP) implant and at 6 months, which they correlated with LBBP electrocardiogram characteristics and lead location. They reported that the LBBP leads (Medtronic 3830; Medtronic, Minneapolis, MN, USA) were stable in 94% of patients, while 6% demonstrated loss of conduction system capture correlating with >2-mm lead displacement on computed tomographic angiography.

Dr. Kaza et al., from Imperial College in London, United Kingdom, presented the results of a stratified analysis of the His Optimised Pacing Evaluated for Heart Failure (HOPE-HF) randomized controlled trial, which randomized patients with left ventricular ejection fractions of ≤40% and P–R intervals of ≥200 ms to His bundle pacing versus no pacing. They assessed the impact of baseline parameters (P–R interval, P–R segment, and acute hemodynamic response) on the trial endpoints of improvements in VO_2_max and Minnesota Living with Heart Failure questionnaire score. In this study, acute hemodynamic response consistently predicted clinical benefits after His bundle pacing.

Congratulations to Dr. Kaza and her colleagues on winning the “Top Abstract” award and to Drs. Ponnusamy and Kielbasa as finalists for their important scientific contributions to the field of physiologic pacing.

We look forward to your attendance at next year’s Ninth Annual Physiology of Pacing Symposium, which is scheduled to be held on October 17, 2025, in Phoenix, Arizona. As a hybrid conference, attendees will have the choice of attending the symposium virtually or as a traditional in-person event.

Sincerely,

Pugazhendhi Vijayaraman, md

Geisinger Heart Institute, Wilkes Barre, PA, USA

Gopi Dandamudi, md

Prisma Health, Greenville, SC, USA

Kenneth A. Ellenbogen, md

Virginia Commonwealth University Health System, Richmond, VA, USA

Gaurav Upadhyay, md

University of Chicago Medicine, Chicago, IL, USA

Roderick Tung, md

Arizona College of Medicine, Banner Health, Phoenix, AZ, USA

